# Supplementary material for: Robust Mendelian randomization in the presence of residual population stratification, batch effects and horizontal pleiotropy
Source: Nat Commun. 2022 Mar 1;13:1093. doi: 10.1038/s41467-022-28553-9 (PMC8888767; doi:10.1038/s41467-022-28553-9)
Supplement: Supplementary file 1 — Supplementary Information [file 41467_2022_28553_MOESM1_ESM.pdf]

Supplementary Information

Robust Mendelian randomization in the presence of residual population stratification, batch effects and horizontal pleiotropy

Carlos Cinelli, Nathan LaPierre, Brian Hill, Sriram Sankararaman, and Eleazar Eskin

Supplementary Note

HDL, LDL and CAD. While High Density Lipoprotein (HDL) levels are generally not considered to have a causal effect on Coronary Artery Disease (CAD), many Mendelian Randomization (MR) studies have reported a statistically significant effect of HDL on CAD[1]. One possible explanation for such results concerns the fact that the genetic variants affecting HDL cholesterol exhibit horizontal pleiotropy through traits such as Low Density Lipoprotein (LDL) cholesterol and Triglycerides[2]. A sensitivity analysis of the MR estimate of the effect of HDL on CAD thus makes for an interesting case study. As a point of contrast, we also perform sensitivity analysis of the MR estimate of the effect of LDL on CAD, which is considered to have a true causal effect[1].

We performed MR and sensitivity analyses of the HDL, LDL, and CAD associations following the same procedures outlined in the main text and Methods section. HDL and LDL were measured in mmol/L (field category IDs 30760 and 30780, respectively). Triglyceride levels (mmol/L) were also obtained in order to include them as a covariate (field cateogry ID: 30870). CAD was defined as the presence of angina or myocardial infarction, following Lyall et al[3]. The data for angina and myocardial infarcation were based on patient touchscreen selections for these conditions when prompted for "Vascular/heart problems diagnosed by doctor" (field category ID: 6150). A polygenic risk score was constructed for unrelated White British participants in the UK Biobank—the same set of participants described in the Methods section. The weights were taken from a previous MR analysis of HDL and LDL performed by Lanktree et al[4] (Supplementary Data 2 and 3), which were obtained based on data by Willer et al[5] as part of the Global Lipids Genetics Consortium.

Table 1 shows the results of standard MR analyses for the effects of HDL and LDL on CAD. All the analyses further adjust for age, sex, genotype batch, assessment centre, and the top 20 principal components. Triglyceride levels were also included, since it can be a potential pleiotropic pathway in both cases. Finally, for the HDL analysis, LDL level was included as a covariate, and vice-versa. In both cases, similar, albeit slightly stronger results were obtained when not controlling for HDL/LDL and Triglycerides.

Henceforth, we call the polygenic risk scores for HDL and LDL as PRS_{HDL} and PRS_{LDL}, respectively. The genetic association of the polygenic risk scores with both exposures turned out to be quite strong. The partial R^2 between PRS_{HDL} and HDL is 7.46%, meaning that, even if a confounder explained 100% of the residual variance of HDL, it would also need to explain at least 7.46% of the residual variance of PRS_{HDL} to fully eliminate the genetic association. Or, equivalently, in terms of pleitropy, this means that, even if a pleitropic effect explained all residual variation of HDL, the polygenic risk score for HDL would need to explain at least 7.46% of this pleoitropic trait to bring down the genetic association to zero. Moreover, the robusness value for the PRS_{HDL}-HDL association is 24.35%, meaning that omitted variables that explain less than 24% both of PRS_{HDL} and of HDL cannot logically make this genetic association statistically insignificant (at the $\alpha = 5\%$ level). For LDL, these numbers were 1.60% for the partial R^2 and 11.63% for the robustness value. Therefore, in both cases, weak instruments seem unlikely to be a problem. We now examine the sensitivity statistics for the genetic associations with the outcome. As explained, these give us the robustness of the MR analysis with respect to the null hypothesis of zero effect. As expected, such associations are more fragile, since the polygenic risk scores were constructed to predict the exposure traits (HDL or LDL) and not the outcome trait (CAD). The robustness value for the association of PRS_{HDL} and CAD was 0.94% and the partial R^2 was 0.018%. Similarly, for PRS_{LDL}, the robustness value was 0.96% and the partial R^2 was 0.018%.

To aid the interpretation of these numbers, we can use the procedures described in the main text and methods section

	Traditional MR		Sensitivity PRS-Outcome		Sensitivity PRS-Exposure	
Exposure	Risk Difference (95% CI)	P-value	Partial R^2	$\text{RV}_{\alpha=0.05}$	Partial R^2	$\text{RV}_{\alpha=0.05}$
HDL	-0.012 (-0.016 to -0.008)	6.7×10^{-11}	0.018%	0.94%	7.46%	24.35%
LDL	0.021 (0.015 to 0.028)	3.3×10^{-10}	0.018%	0.96%	1.60%	11.63%

Supplementary Table 1. Traditional Mendelian Randomization (MR) results and sensitivity analyses for the effect of High Density Lipoprotein (HDL) and Low Density Lipoprotein (LDL) on Coronary Artery Disease (CAD). P-values correspond to two-sided t-tests in a two-stage least squares regression. No multiple testing corrections were performed. CI, Confidence Interval; PRS = Polygenic Risk Score; RV = Robustness Value.

to bound the maximum strength of unobserved variables, if they had the same (or a multiple of the) strength of observed variables. Tables 2 and 3 show the maximum explanatory power that unobserved variables would have if they were as strong as: (i) the first 20 principal components (PCs), (ii) observed batch effects (Batch+Centre), and (iii) LDL (or HDL) and Triglyceride levels (as a benchmark for potential residual pleiotropy). Note, for both analyses, the genetic associations with CAD would survive residual pleiotropy or batch effects as strong as the observed PCs and batch indicators, since the bounds for all traits are below the robustness values for the respective genetic associations, reported in Supplementary Table 1. The situation, however, is somewhat different for residual pleiotropy. Starting with HDL, note that unobserved variables as strong as the combined explanatory power of LDL and Triglycerides could explain 4.20% of CAD and 1.04% of PRS_{HDL}. Since both numbers are above the robustness value of 0.94%, this means residual pleitropy with such strength could be sufficiently strong to explain away the causal effect of HDL on CAD. In contrast, Supplementary Table 3 shows the corresponding bounds on the partial R^2 of residual pleitropy as strong as HDL + Triglycerides, which amount to 0.79% and 0.81%. As both below the robustness value for the effect of LDL (0.96%), this means that such residual biases would not be sufficient to revert the effect of LDL on CAD.

These bounding exercises can be further refined with the contour plots shown of Supplementary Figure 1. Again, we start with the HDL contours (Figure 1a). We see that, for HDL, the unadjusted t-statistic is -6.7. Additionally, confounding with 18 times the strength of the top 20 principal components, or 8 times the strength of the observed batch indicators, would not bring the t-statistic to the critical threshold of -1.96. However, pleitropic effects as strong as LDL+Triglycerides could cause the adjusted t-statistic to move as far as (positive) 3.92, meaning that a confounder even a fraction as strong as LDL+Triglycerides could not only explain away but revert the sign of the effect of HDL on CA. Turning to the LDL contours (Figure 1b), we observe that the MR estimate is robust to confounders up to 6 times the strength of the first 20 principal components or 10 times the batch effects. Notably, and in contrast with the HDL plot, this MR estimate is also robust to residual pleitropy as strong as HDL+Triglycerides. Taken together, the sensitivity analysis thus indicates that al-

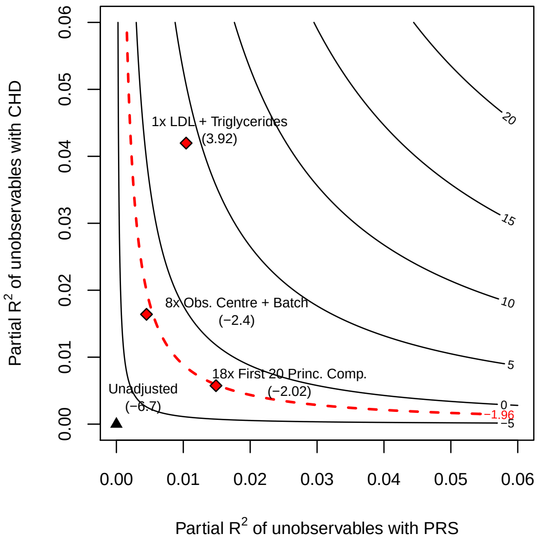

(a) Sensitivity contour for HDL.

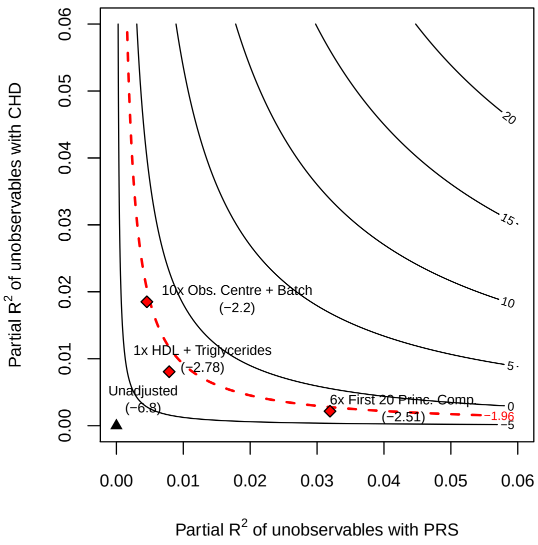

(b) Sensitivity contour for LDL.

Supplementary Figure 1. Sensitivity contours for the null hypothesis of zero effect. (a) Sensitivity contour for HDL. (b) Sensitivity contour for LDL.

	Bound partial R^2 with genetic IV	Bound partial R^2 with trait	
W as strong as	PRS_{HDL} (Genetic IV)	HDL (Exposure)	CAD (Outcome)
1 x PCs	0.08%	0.03%	0.03%
1 x Batch+Centre	0.06%	0.38%	0.21%
1 x LDL+Triglycerides	1.04%	21.75%	4.20%

Supplementary Table 2. Bounds on the maximum explanatory power of ***W*** (partial R^2) for the HDL-CAD MR analysis, if it were as strong as: (i) 20 leading genomic principal components (1 x PCs); (ii) observed batch and centre (1 x Batch+Centre); and, (iii) LDL and Triglyceride levels (1 x LDL+Triglycerides). CAD = Coronary Artery Disease; HDL = High Density Lipoprotein; IV,= - Instrumental Variable; LDL = Low Density Lipoprotein; PCs = Principal Components; PRS = Polygenic Risk Score.

$\boldsymbol{W}$ as strong as	Bound partial R^2 with genetic IV<br>PRS_{LDL} (Genetic IV)	Bound partial R^2 with trait<br>LDL (Exposure)	 CAD (Outcome)
1 x PCs	0.53%	0.04%	0.04%
1 x Batch+Centre	0.05%	0.20%	0.19%
1 x HDL+Triglycerides	0.79%	10.18%	0.81%

Supplementary Table 3. Bounds on the maximum explanatory power of $\boldsymbol{W}$ (partial R^2) for the LDL-CAD MR analysis, if it were as strong as: (i) 20 leading genomic principal components (1 x PCs); (ii) observed batch and centre (1 x Batch+Centre); and, (iii) HDL and Triglyceride levels (1 x HDL+Triglycerides). CAD = Coronary Artery Disease; HDL = High Density Lipoprotein; IV = Instrumental Variable; LDL = Low Density Lipoprotein; PCs = Principal Components; PRS = Polygenic Risk Score.

though both the HDL-CAD and LDL-CAD MR estimates are relatively robust to confounding from population structure or batch effects, the LDL-CAD estimate is relatively more resilient to pleiotropy.

As a final remark, we caution readers that the methods discussed in this paper have been developed for performing sensitivity analyses of the traditional MR estimand, namely, the ratio of the two OLS coefficients:

$$\tau := \frac{\beta_{YZ|\mathbf{XW}}}{\beta_{DZ|\mathbf{XW}}} \tag{1}$$

Note, however, that CAD is a binary trait, and thus this may not be the best approach for this analysis. On the other hand, since our main focus is on assessing the null hypothesis of zero effect, and since the genetic effects are small, the results of logistic regression turn out to be similar to OLS. Thus, here, it may be the case that this difference is immaterial. This is not a formal proof, though—formal extensions of the sensitivity tools of this paper to non-linear models is currently under work.

E-Values for the BMI study. In our running example, one obtains the E-value[6] approximations for the standardized linear regression coefficients of 1.15 (1.13 for the 95% CI) for DBP, 1.07 (1.03 for the 95% CI) for Deprivation, and 1.50 (1.49 for the 95% CI) for the first stage (PRS-BMI association). If we take the rule-of-thumb suggested in some papers[6,7] to judge what is a "big" or "small" E-value, we would conclude that "modest confounding" would be sufficient to explain away the genetic associations with BMI or DBP. The analysis in the main text, however, reveals that it does take some fairly strong confounding to do so. The problem here is that the risk ratio scale is not natural in this setting, and one cannot simple "transfer" knowledge of what is a "big" or "small" risk ratio from other fields directly to MR, without further context. Here, the partial R^2 parameterization has the benefit of speaking directly to quantities researchers in these areas are used to thinking about. For an illustration, consider notions such as heritability, or simply the fact that many simulations in MR studies actually parameterize the strength of genetic effects in terms of partial R^2. Nevertheless, here we reinforce the statement of Cinelli and Hazlett[8] with respect to the RV and the E-value—researchers should be aware of both options, as sometimes one metric can be easier to reason about than the other. Finally, note currently there are no formal benchmarking procedures to leverage observed covariates to bound the strength of the unobserved confounder under the E-value framework. Recent attempts to fill this gap[9] rely on what is known as "informal benchmarking" (see Cinelli and Hazlett [8, Sections 4.4 and 6.2]), and may lead users to erroneous conclusions. In fact, this is a problem that can also affect "bias components" plots[10,11] whenever they are used to extrapolate claims about unmeasured confounders, as we discuss next.

Bias components plots. "Bias components" plots[10,11] have been proposed in Epidemiology as a useful tool to understand and decompose the difference between an IV estimate including and excluding observed variables, and contrast this to usual "adjustment" estimates, again including and excluding observed variables. These tools, however, do not provide formal sensitivity analysis due to unmeasured confounding, as we do in the paper. Moreover, while there are claims that bias components plots could be *"informative to potential bias when the type of unmeasured confounding is expected to be similar to what we observe,"* [10, p. 503] this is not necessarily true, even in the ideal case of having unmeasured confounder that is identical to the measured confounder in its associations with all other variables of the system. To witness, consider the model below (interpret this as pseudocode to create the data, "expit" is the inverse of the logit function):

$$W \leftarrow \text{Binomial}(p = 0.5) \tag{2}$$
$$X \leftarrow \text{Binomial}(p = 0.5) \tag{3}$$
$$Z \leftarrow \text{Binomial}(p = \text{expit}(W + X)) \tag{4}$$
$$D \leftarrow \text{Binomial}(p = \text{expit}(Z + W + X)) \tag{5}$$
$$Y \leftarrow \text{Normal}(\mu = X + W, \sigma^2 = 1) \tag{6}$$

Where, as in the main text, Z is the instrument, D the exposure, Y the outcome, and X and W are the confounders. Note how the observed confounder X and the unobserved confounder W are completely symmetric. Also note the true causal effect of D on Y is zero. Therefore, we would like the result of a sensitivity analysis to tell us that an unmeasured variable W "as strong as" the measured variable X would be sufficient to overturn the observed results. Yet, if one uses the "observed

	Proportion of rejections of the null ($\alpha = 5\%$)						Critical k		$RV_{\alpha=0.05}$	
Relative strength of W	IVW	PRESSO	Egger	GENIUS	MBE	Mix	5th	95th	5th	95th
2	100%	100%	86%	100%	100%	75%	1.1	1.9	1.0%	1.3%
3	100%	100%	99%	100%	100%	74%	2.0	2.8	1.5%	2.0%
4	100%	100%	100%	100%	100%	75%	2.8	3.9	2.2%	2.7%

Supplementary Table 4. Simulation varying the strength of unobserved variable W relative to the observed variable X. Sample size of $450,000$ and $\tau = 0$. All methods are run with default parameter choices. RV = Robustness Value.

bias" due to X to infer the bias due to the unmeasured variable W (without proper adjustment) this leads to the incorrect conclusion that an "unmeasured confounder W similar to the measured confounder X" would not be sufficiently strong to explain away the IV estimate, when in fact it would. The main problem here is collider bias; although X and W are independent, they become dependent after conditioning either on D or on Z, and thus the observed association of X with Y is dampened. For this reason we recommend using formal bounds on the unmeasured confounder, such as the ones we discuss in the paper. Further discussion of this problem can be found in Cinelli and Hazlett [8, Sections 4.4 and 6.2].

Simulations varying the relative strength of W. We performed an additional simulation scenario in which we vary the relative strength of the unobserved variable W to be two, three, or four times as strong as the strength of the observed variable X. We do this by, in the data generating process of Equations 1 to 3, scaling the coefficients ϕ and γ by $\sqrt{k}$, where k equals 2, 3 or 4. The result are shown in Supplementary Table 4.

Note how the sensitivity analysis correctly reveals that it would take a an unobserved confounder W about 1 to 2, 2 to 3, or 3 to 4 times stronger than X to make the MR estimate statistically insignificant (since we are considering statistical significance, the "sample" k is more conservative than the population k). In other words, the sensitivity analysis is telling the correct story to the researcher: either there exists some confounder about k times as strong as X, or, if such strengths are not plausible (we know they are in the simulation), then there must be some true causal effect in order to explain the observed association.

Additional simulations for MR-GENIUS. MR-GENIUS[12] operates under the assumption that the error terms of the first stage regression are heteroscedastic. The DGP in the main text does not satisfy this assumption. Therefore, here we perform an additional simulation in which we introduce heteroskedasticity, and investigate how MR-GENIUS behaves under various strengths of heteroskedasticity. We parameterize the level of heteroskedasticity by multiplying the error term $\varepsilon_{i,D}$ of the treatment equation by $\sigma \times \sum_{j=1}^{J} \kappa_j G_{ij}$ where κ_j is draw from a uniform distribution from 0.01 and 0.05 and σ is a scalar further controlling the strength of heteroskedaticity. The results are shown in Supplementary Table 5. We find that MR-GENIUS still outputs high false positive rates when the level of heteroskedasticity is low—for instance, the false positive rate was stil 25% when $\sigma = 1$, and only reached values close to the nominal level when $\sigma = 3$.

Alternative DAG structures. Recall our focus is on the sensitivity analysis of the IV estimand, namely, the ratio:

$$\tau := \frac{\beta_{YZ|\mathbf{XW}}}{\beta_{DZ|\mathbf{XW}}} \tag{7}$$

This IV estimand can have different causal interpretations, depending on the assumptions the researcher is wiling to defend[13–16]. For instance, under certain assumptions of effect homogeneity, τ can be interpreted as the average treatment effect. In the binary setting, under the assumption of monotonicity and other functional restrictions, τ may be interpreted as a weighted average of local average treatment effects. Or, if one is interested in testing the sharp null hypothesis of zero effect for all individuals, effect homogeneity holds by definition (in all scales). So long as the researcher's target of inference is the IV estimand of Equation 7, all sensitivity results presented here can be used, regardless of the particular set of causal assumptions that justified the target of inference.

For this reason, although violations of the IV assumptions can arise due to very different structures, the mechanics of the sensitivity analyses remains the same. For concreteness, Supplementary Figure 2 shows some examples discussed in the literature[17,18] (for simplicity, suppose all structural equations are linear).

- Supplementary Figure 2a: suppose we have a time-varying exposure D_1 and D_2 that affects Y. If the re-

	Strength of heteroskedacity σ						
	0	0.25	0.5	0.75	1	2	3
False Positives	100%	99.9%	89.7%	51.6%	26.7%	7.5%	5.6%

Supplementary Table 5. False positives for MR-GENIUS when varying the strength of heteroskedasticity as parameterized by σ. Sample size fixed at $N = 100,000$, true causal effect set at $\tau = 0$.

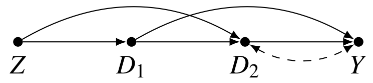

(a) Time-varying treatment[17].

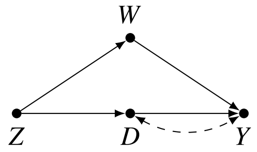

(b) Traditional pleiotropy[17].

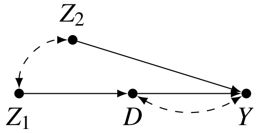

(c) Linkage disequilibrium[17].

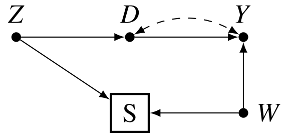

(d) Selection bias ("time zero misalignment"[18]).

Supplementary Figure 2. Different IV models with alternative substantive interpretations, yet resulting in the same procedure for sensitivity analysis. (a) Time-varying treatment. (b) Traditional pleitropy. (c) Linkage Disequilibrium. (d) Selection bias.

searcher is interested in the (contemporaneous) average treatment effect of D_2 on Y, the conditional set $W = \{D_1\}$ is sufficient for making Z a valid genetic instrument. Thus, if D_1 is not measured, the sensitivity parameters here would consist of postulating hypothetical strengths for how much residual variation Z explains of D_1, and how much residual variation D_1 explains of Y.

- Supplementary Figure 2b: suppose we are interested in average treatment effect of D on Y, but the genetic instrument Z also affects trait W. Here W is again sufficient to make Z a valid instrument, and the sensitivity parameters would have the same interpretation as before.

- Supplementary Figure 2c: suppose we are interested in the average treatment effect of D on Y, but the genetic instrument Z_1 is in linkage disequilibrium with another variant Z_2. As before, if Z_2 is not measured, one can perform sensitivity analysis by positing hypothetical strengths of the linkage disequilibrium (as parameterized by the partial correlation of Z_1 and Z_2), and how much residual variation the genetic variant Z_2 explains of Y.

- Supplementary Figure 2d: suppose we are interested in the average treatment effect of D on Y on a study sample $S = 1$, but both the genetic instrument Z and the unobserved variable W affect the indicator of whether the participant is selected to the study. Note Z is not a valid instrument for the causal effect of D on Y for the selected sample $S = 1$. But further note that Z can be made a valid instrument when we *further* condition on W. This is precisely the case of our setup, and thus one can use all of our tools for performing sensitivity analysis in this setting.

In sum, there are many ways in which the violations of IV assumptions can happen, and as likely as many ways one could posit hypothetical variables $\mathbf{W}$ that would then render Z a valid genetic instrument. By design, our sensitivity analysis is agnostic to which specific causal assumptions one makes, so long as the target of inference is the IV estimand of Equation 7.

Finally, one may wonder how is it that the same approach can be used for such different target effects—after all, if the target effects are different, shouldn't the biases also be different? The important thing to notice here is that, although we are using the same set of analytical *tools* for assessing these biases, the magnitude of the biases will not be the same when contemplating different target causal quantities. This happens because the set of (hypothetical) unobserved variables that should be part of the conditioning set $\boldsymbol{W}$ will, in general, differ for each target causal effect of interest. Moreover, further note that the robustness of an observed association to the inclusion of omitted variables is a *property of the data*, and it is independent both from a possible causal interpretation of that association, and from whether omitted variables with such strengths are likely to exist.

Supplementary References

1. Morrison, J., Knoblauch, N., Marcus, J. H., Stephens, M. & He, X. Mendelian randomization accounting for correlated and uncorrelated pleiotropic effects using genome-wide summary statistics. *Nat. genetics* **52**, 740–747 (2020).

2. Voight, B. F. *et al.* Plasma hdl cholesterol and risk of myocardial infarction: a mendelian randomisation study. *The Lancet* **380**, 572–580 (2012).

3. Lyall, D. M. *et al.* Association of body mass index with cardiometabolic disease in the uk biobank: a mendelian randomization study. *JAMA cardiology* **2**, 882–889 (2017).

4. Lanktree, M. B., Thériault, S., Walsh, M. & Paré, G. Hdl cholesterol, ldl cholesterol, and triglycerides as risk factors for ckd: a mendelian randomization study. *Am. J. Kidney Dis.* **71**, 166–172 (2018).

5. Willer, C. J. *et al.* Discovery and refinement of loci associated with lipid levels. *Nat. genetics* **45**, 1274 (2013).

6. VanderWeele, T. J. & Ding, P. Sensitivity analysis in observational research: introducing the e-value. *Annals internal medicine* **167**, 268–274 (2017).

7. Swanson, S. A. & VanderWeele, T. J. E-values for mendelian randomization. *Epidemiology* **31**, e23–e24 (2020).

8. Cinelli, C. & Hazlett, C. Making sense of sensitivity: extending omitted variable bias. *J. Royal Stat. Soc. Ser. B* **82**, 39–67, DOI: 10.1111/rssb.12348 (2020).

9. D'Agostino McGowan, L. & Greevy Jr, R. A. Contextualizing e-values for interpretable sensitivity to unmeasured confounding analyses. *arXiv e-prints* arXiv–2011 (2020).

10. Jackson, J. W. & Swanson, S. A. Toward a clearer portrayal of confounding bias in instrumental variable applications. *Epidemiology* **26**, 498 (2015).

11. Davies, N. M. Commentary: an even clearer portrait of bias in observational studies? *Epidemiol. (Cambridge, Mass.)* **26**, 505 (2015).

12. Tchetgen, E. T., Sun, B. & Walter, S. The genius approach to robust mendelian randomization inference. *Stat. Sci.* **36**, 443–464 (2021).

13. Angrist, J. D., Imbens, G. W. & Rubin, D. B. Identification of causal effects using instrumental variables. *J. Am. statistical Assoc.* **91**, 444–455 (1996).

14. Angrist, J. & Pischke, J.-S. *Mostly harmless econometrics: an empiricists guide* (Princeton: Princeton University Press, 2009).

15. Pearl, J. *Causality* (Cambridge university press, 2009).

16. Swanson, S. A., Hernán, M. A., Miller, M., Robins, J. M. & Richardson, T. S. Partial identification of the average treatment effect using instrumental variables: review of methods for binary instruments, treatments, and outcomes. *J. Am. Stat. Assoc.* **113**, 933–947 (2018).

17. VanderWeele, T. J., Tchetgen, E. J. T., Cornelis, M. & Kraft, P. Methodological challenges in mendelian randomization. *Epidemiology* **25**, 427 (2014).

18. Swanson, S. A. A practical guide to selection bias in instrumental variable analyses. *Epidemiology* **30**, 345–349 (2019).
