# Supplementary material for: Robust Mendelian randomization in the presence of residual population stratification, batch effects and horizontal pleiotropy
Source: Nat Commun. 2022 Mar 1;13:1093. doi: 10.1038/s41467-022-28553-9 (PMC8888767; doi:10.1038/s41467-022-28553-9)
Supplement: Supplementary file 3 — Description of Additional Supplementary Files [file 41467_2022_28553_MOESM3_ESM.pdf]

Description of Additional Supplementary Files

File Name: Supplementary Data 1
Description: TSV file containing the SNPs used to create the BMI polygenic risk score, along with the effect allele and the effect sizes, adapted from Lyall et al [1].

File Name: Supplementary Data 2
Description: TSV file containing the SNPs used to create the HDL polygenic risk score, along with the effect allele and the effect sizes, adapted from Lanktree et al [2] and the Global Lipids Genetics Consortium [3].

File Name: Supplementary Data 3
Description: TSV file containing the SNPs used to create the LDL polygenic risk score, along with the effect allele and the effect sizes, adapted from Lanktree et al [2] and the Global Lipids Genetics Consortium [3].

References:

[1] Lyall DM, Celis-Morales C, Ward J, Iliodromiti S, Anderson JJ, Gill JM, Smith DJ, Ntuk UE, Mackay DF, Holmes MV, Sattar N. Association of body mass index with cardiometabolic disease in the UK Biobank: a Mendelian randomization study. JAMA cardiology. 2017 Aug 1;2(8):882-9.

[2] Lanktree MB, Thériault S, Walsh M, Paré G. HDL cholesterol, LDL cholesterol, and triglycerides as risk factors for CKD: a Mendelian randomization study. American Journal of Kidney Diseases. 2018 Feb 1;71(2):166-72.

[3] Willer CJ, Schmidt EM, Sengupta S, Peloso GM, Gustafsson S, Kanoni S, Ganna A, Chen J, Buchkovich ML, Mora S, Beckmann JS. Discovery and refinement of loci associated with lipid levels. Nature genetics. 2013 Nov;45(11):1274.
