# Supplementary material for: Robust Mendelian randomization in the presence of residual population stratification, batch effects and horizontal pleiotropy
Source: Nat Commun. 2022 Mar 1;13:1093. doi: 10.1038/s41467-022-28553-9 (PMC8888767; doi:10.1038/s41467-022-28553-9)
Supplement: Supplementary file 7 — Reporting Summary [file 41467_2022_28553_MOESM7_ESM.pdf]

nature portfolio

Corresponding author(s): Carlos Cinelli

Last updated by author(s): Nov 2, 2021

Reporting Summary

Nature Portfolio wishes to improve the reproducibility of the work that we publish. This form provides structure for consistency and transparency in reporting. For further information on Nature Portfolio policies, see our Editorial Policies and the Editorial Policy Checklist.

Statistics

For all statistical analyses, confirm that the following items are present in the figure legend, table legend, main text, or Methods section.

n/a	Confirmed	
☒	☐	The exact sample size (*n*) for each experimental group/condition, given as a discrete number and unit of measurement
☐	☒	A statement on whether measurements were taken from distinct samples or whether the same sample was measured repeatedly
☐	☒	The statistical test(s) used AND whether they are one- or two-sided<br>*Only common tests should be described solely by name; describe more complex techniques in the Methods section.*
☐	☒	A description of all covariates tested
☐	☒	A description of any assumptions or corrections, such as tests of normality and adjustment for multiple comparisons
☐	☒	A full description of the statistical parameters including central tendency (e.g. means) or other basic estimates (e.g. regression coefficient) AND variation (e.g. standard deviation) or associated estimates of uncertainty (e.g. confidence intervals)
☐	☒	For null hypothesis testing, the test statistic (e.g. *F*, *t*, *r*) with confidence intervals, effect sizes, degrees of freedom and *P* value noted<br>*Give P values as exact values whenever suitable.*
☒	☐	For Bayesian analysis, information on the choice of priors and Markov chain Monte Carlo settings
☒	☐	For hierarchical and complex designs, identification of the appropriate level for tests and full reporting of outcomes
☐	☒	Estimates of effect sizes (e.g. Cohen's *d*, Pearson's *r*), indicating how they were calculated

Our web collection on statistics for biologists contains articles on many of the points above.

Software and code

Policy information about availability of computer code

Data collection

No software was used for data collection.

Data analysis

All data was analyzed using R 3.5.1.

A custom R package, mr-sensemakr, was written to implement the methods described in the paper, available at: https://github.com/carloscinelli/mrsensemakr.

Replication code for the simulation can also be found at https://github.com/carloscinelli/mrsensemakr.

mr-sensemakr builds upon the R package sensemakr, available at: https://github.com/carloscinelli/sensemakr .

Additional R packages were used for running simulations. These are:

MR-PRESSO: https://github.com/rondolab/MR-PRESSO

MendelianRandomization: https://cran.r-project.org/package=MendelianRandomization

paramtest: https://cran.r-project.org/package=paramtest

MRMix: https://github.com/gqi/MRMix

genius: https://github.com/bluosun/MR-GENIUS

snow: https://cran.r-project.org/package=snow

For manuscripts utilizing custom algorithms or software that are central to the research but not yet described in published literature, software must be made available to editors and reviewers. We strongly encourage code deposition in a community repository (e.g. GitHub). See the Nature Portfolio guidelines for submitting code & software for further information.

Data

Policy information about availability of data

All manuscripts must include a data availability statement. This statement should provide the following information, where applicable:

- Accession codes, unique identifiers, or web links for publicly available datasets
- A description of any restrictions on data availability
- For clinical datasets or third party data, please ensure that the statement adheres to our policy

All genotype and phenotype data are available by application from the UKBB at: http://www.ukbiobank.ac.uk/

Field-specific reporting

Please select the one below that is the best fit for your research. If you are not sure, read the appropriate sections before making your selection.

☒ Life sciences ☐ Behavioural & social sciences ☐ Ecological, evolutionary & environmental sciences

For a reference copy of the document with all sections, see nature.com/documents/nr-reporting-summary-flat.pdf

Life sciences study design

All studies must disclose on these points even when the disclosure is negative.

Sample size	The work described in this manuscript uses data from previous studies using data from the UK Biobank (UKBB). Therefore, we do not have control over the sample size for these studies. The data consists of a cohort of 503,325 British people aged 37-73 years. More details of the UKBB data can be found at http://www.ukbiobank.ac.uk/
Data exclusions	We filtered the data to only include people with self-reported white British ancestry who were not closely related, (e.g. no first, second, or third degree relatives), as defined by pairs of individuals who had a kinship coefficient <(1/2)^(9/2).
Replication	All results presented in the paper can be replicated with code available on our website https://github.com/carloscinelli/mrsensemakr.
Randomization	We did not perform any randomized experiments that involves assigning individuals to groups.
Blinding	We did not perform any experiments that involves assigning individuals to groups.

Reporting for specific materials, systems and methods

We require information from authors about some types of materials, experimental systems and methods used in many studies. Here, indicate whether each material, system or method listed is relevant to your study. If you are not sure if a list item applies to your research, read the appropriate section before selecting a response.

Materials & experimental systems

n/a	Involved in the study	
☒	☐	Antibodies
☒	☐	Eukaryotic cell lines
☒	☐	Palaeontology and archaeology
☒	☐	Animals and other organisms
☐	☒	Human research participants
☒	☐	Clinical data
☒	☐	Dual use research of concern

Methods

n/a	Involved in the study	
☒	☐	ChIP-seq
☒	☐	Flow cytometry
☒	☐	MRI-based neuroimaging

Human research participants

Policy information about studies involving human research participants

Population characteristics	See above. Details of the UKBB at http://www.ukbiobank.ac.uk/
Recruitment	See above. Details of the UKBB at http://www.ukbiobank.ac.uk/

Ethics oversight	See above. Details of the UKBB at http://www.ukbiobank.ac.uk/

Note that full information on the approval of the study protocol must also be provided in the manuscript.
